# The role of metabolic syndrome in osteoarthritis development: Is obesity the key driver?

**DOI:** 10.1016/j.ocarto.2025.100739

**Published:** 2025-12-31

**Authors:** Dell’Isola Andrea, Magnusson Karin, Vinblad Johanna, Lohmander L. Stefan, Englund Martin, Kiadaliri Ali

**Affiliations:** a Faculty of Medicine, Department of Clinical Sciences Lund, Orthopedics, Clinical Epidemiology Unit, Lund University, Lund, Sweden; b Norwegian Institute of Public Health, Oslo, Norway; c Department of Orthopaedics, Institute of Clinical Sciences, Sahlgrenska Academy, University of Gothenburg, Gothenburg, Sweden; d Faculty of Medicine, Department of Clinical Sciences Lund, Orthopedics, Lund University, Lund, Sweden

**Keywords:** Osteoarthritis, Metabolic syndrome, Obesity

## Abstract

**Objective:**

Investigate the role of metabolic syndrome and obesity in incident osteoarthritis (OA).

**Methods:**

Prospective cohort study with up to 11 years follow-up. From a general population cohort of 28,786 individuals from Sweden aged 45 to 75, we selected individuals without OA diagnosis and without joint pain during the year prior to the baseline. Metabolic syndrome was defined at baseline by the presence of at least three out of five components: elevated waist circumference (WC), elevated plasma triglycerides, reduced HDL-cholesterol, elevated blood pressure, and hyperglycaemia. Individuals were followed from baseline until incident OA diagnosis in any joint (outcome), death, relocation outside Region Skåne/Region Uppsala, or December 31, 2022. Associations between metabolic syndrome and OA incidence were estimated using parametric survival models with restricted cubic splines and adjusted for sex, age, education, immigration status, marital status, physical activity, and diet score.

**Results:**

Among the 10,633 individuals, 1167 (11 %) received an OA diagnosis (median follow-up 8.6 years). Metabolic syndrome was associated with an increased risk of OA (hazard ratio [HR] 1.17, confidence intervals [1.04, 1.33]). Elevated WC alone was associated with a similar risk of OA (HR 1.42 [1.06, 1.90]), while metabolic syndrome without elevated WC did not show a conclusive association (HR 1.02 [0.73, 1.43]). Substituting WC with BMI led to similar results.

**Conclusions:**

Metabolic syndrome leads to an increased risk of developing OA even in individuals free of joint pain at baseline. The association is largely driven by obesity, which underscores the importance of weight management to mitigate OA development.

## 1 Introduction

Osteoarthritis (OA) is one of the most burdensome and fastest-growing causes of disability worldwide, a rise driven in part by lifestyle and environmental factors, including low physical activity and high-calorie diet, leading to overweight or obesity [1].

OA and metabolic syndrome—a cluster of central obesity, hypertension, raised triglycerides, low high-density lipoprotein cholesterol (HDL-C), and glucose intolerance–often coexist. While shared lifestyle and environmental risk factors partially explain this association, growing evidence suggests that the connection extends beyond these factors. Biomechanical joint overload from increased body mass and metabolic dysregulation creates a pro-inflammatory and catabolic environment. This environment accelerates OA progression and increases pain, supporting the existence of an OA phenotype driven by metabolic imbalance [[2], [3], [4], [5], [6]].

Previous epidemiological studies have attempted to study the link between metabolic syndrome and OA incidence, showing contrasting results [[7], [8], [9], [10], [11], [12], [13], [14], [15]]. These contrasting results can be partly explained by data constraints, including the lack of systematically reported primary care data—where OA is most often diagnosed—forcing most studies to rely on joint replacements to establish incident OA. While both OA diagnosis and joint replacement have limitations when used to ascertain OA incidence, the latter misclassifies the individuals who develop OA but do not develop symptoms severe enough to warrant a replacement. Moreover, metabolic conditions can be relative contraindications to surgery, complicating the interpretation of estimates [16].

Another important factor often overlooked in previous studies is that OA can be a risk factor for metabolic syndrome, likely through reduced physical activity and associated weight gain [17]. Given OA's slow development and the fact that symptoms often precede a formal diagnosis by several years, failing to account for joint symptoms at the start of follow-up may lead to reverse causality bias. In such cases, the higher risk of developing OA among people with metabolic syndrome may reflect the presence of undiagnosed OA at the start of follow-up. Finally, the effect of metabolic syndrome may vary across joints, with the knee often shown to be more susceptible to metabolic risk factors and abnormal loading due to obesity than the hip [10,15].

In this study, we attempted to solve these problems by investigating the role of metabolic syndrome on incident clinical OA (diagnosed either in primary or specialist care) in a population sample of individuals free of both diagnosed or self-reported OA and self-reported joint symptoms at baseline. We then attempted to disentangle the role of each metabolic syndrome component, with particular attention to waist circumference (WC), by analysing the components separately and contrasting the presence of WC alone and in combination with other metabolic syndrome components. Finally, we substituted BMI for WC and repeated all the analyses, comparing the estimates to understand which obesity measure is best associated with the risk of OA.

## 2 Methods

### 2.1 Ethical approval

The current study was approved by the Swedish Ethical Review Authority (application numbers: 2022-00174-01; 2025-07-15).

### 2.2 Data sources

We used data from the Epidemiology for Health (EpiHealth) cohort study initiated in 2011 and conducted in the Swedish cities of Malmö and Uppsala [18]. The EpiHealth cohort includes a sample of participants aged 45 to 75 with a permanent address in Sweden and a Swedish civic registration number selected from the official registries and/or commercial address databases employing a randomisation procedure to ensure that all age groups and both genders are equally represented in the main study. The individuals who agreed to participate answered an online survey followed by a visit to a test centre between 2011 and 2018. Individuals could respond to the questionnaire online from home or through a computer available at the test centre. Using encrypted personal identification numbers, we linked data from EpiHealth with register data from the Population Register (1998–2022), the National Patient Register (1987–2022), the Prescribed Drug Register (2005–2022), the Causes of Death Register (2011–2022), the Longitudinal Integration Database for Health Insurance and Labour Market Studies (LISA) and the regional primary healthcare registers (2006–2022) from Skåne (covering Malmö population) and Uppsala. We extracted data on place of residence at the end of each year from the Population Register and date of death from the Causes of Death Register. Data on education, immigration status and marital status were extracted from LISA. From the National Patient Register and regional primary healthcare registers, we collected data on date of visit and diagnoses recorded according to the International Classification of Diseases (ICD)-10 system for all in-person primary care, secondary outpatient care, and tertiary inpatient care visits. We extracted data on prescriptions and dispensing dates, dispensed medications, and Anatomical Therapeutic Chemical (ATC) codes from the Prescribed Drug Register. This study is reported as per the Reporting of Studies Conducted using Observational Routinely-Collected Data (RECORD) guideline [19] (S1 Table).

### 2.3 Participants

**Fig. 1:**
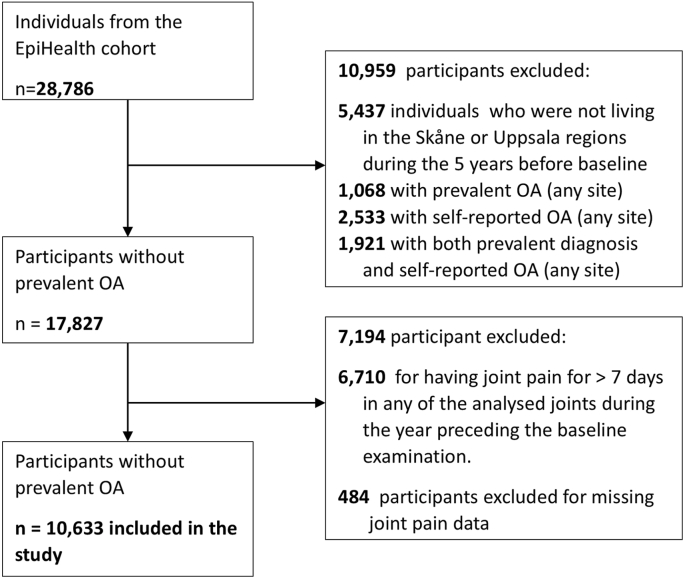
Study flowchart.

We included all individuals from the EpiHealth cohort (n = 28,786) from the date of their baseline examination [18]. We excluded 5437 individuals who were not living in the Skåne or Uppsala regions during the 5 years before baseline (i.e. visit to the test centre for data collection). This criterion was applied to maximise our capacity to determine the presence of prevalent OA among participants. In addition, we excluded those with a previous OA, as indicated by ICD-10 codes M15–M19, which cover generalised OA (M15), hip (M16), knee (M17), first carpometacarpal joint (M18), and other or unspecified sites (M19) (n = 1068). Knee OA diagnosis and specialist care diagnoses in Swedish registers have previously been validated, showing high positive predictive values [20,21]. We also excluded those who self-reported OA by responding “yes” to the question, “Has a doctor ever told you that you have or have had any of the following diseases?” (n = 2533), as well as individuals who met both criteria (n = 1921), for a total of 5522 excluded participants with prevalent OA. Additionally, we excluded 6710 individuals who reported joint pain for >7 days in any of the analysed joints during the year preceding the baseline examination. This was done to reduce the risk of individuals with OA being included in the study and biasing the estimate (i.e. reverse causality). We finally excluded 484 persons with missing responses to joint pain questions (Fig. 1).

### 2.4 Definition of metabolic syndrome and its components

The presence of metabolic syndrome (yes/no) was defined according to consensus by professional societies, which requires the presence of a minimum of 3 out of the following 5 components [22].

1. *Elevated waist circumference* (WC) was defined as ≥94 cm in males or ≥80 cm in females.
2. *Elevated triglycerides* were defined as a plasma triglyceride level ≥150 mg/dL (1.7 mmol/L) or the use of anti-triglyceride medication (ATC code: C108).
3. *Reduced* HDL-C was defined as plasma HDL <40 mg/dL (1.03 mmol/L) in males or < 50 mg/dL (1.29 mmol/L) in females or drug treatment for reduced HDL-C (ATC codes: C10AA, C10AC, C10AX).
4. *Elevated blood pressure* was defined as systolic blood pressure (SBP) ≥130 mm Hg or diastolic blood pressure ≥85 mm Hg or antihypertensive drug treatment (ATC codes: C02, C03, C07, C08, C09) in a patient with a history of hypertension (ICD-10 codes: I10).
5. *Hyperglycemia* was defined as fasting blood glucose ≥100 mg/dL (5.6 mmol/L) or a prior diagnosis of type 2 diabetes (ICD-10 codes: E10.0, E11, E12; E13, E14, R7) or drug treatment for elevated glucose (ATC code: A10).

### 2.5 Outcome and follow-up

The outcome was a principal diagnosis of OA (i.e. OA was the main reason for healthcare contact) at primary, specialist or inpatient care. We used ICD-10 codes to identify persons with any OA (M15-M19). Everyone was followed from the date of visiting a test centre for physical examination (start of follow-up, between 2011 and 2018) until an OA diagnosis at any joint (outcome) or other censoring events (i.e. death, relocation outside Skåne/Uppsala regions, or December 31, 2022), whichever came first. Thus, the follow-up time could be up to 11 years.

### 2.6 Covariates

Based on prior evidence and data available, we included age, sex, level of education (0–9 years, 10–12 years, 13+ years), immigration status (born in Sweden: yes/no), marital status (not married, previously married, married), physical activity and diet as potential confounders. Data on age, sex, education, immigration status and marital status were extracted from the registers. Physical activity during leisure time was self-reported by the participants answering a seven-point question in the survey, ranging from hardly any physical activity to rigorous regular exercises (1–7). The diet was assessed by computing the modified Mediterranean diet (mMED) covering intake of eight groups of food/drink: fruit and vegetables, legumes and nuts, non-refined or high-fiber grains, fermented dairy products, fish, red or processed food, use of olive or rapeseed oil and moderate alcohol consumption, ranging from 0 to 8 [23].

### 2.7 Data analysis

We report baseline characteristics as mean and standard deviation [SD] and numbers (proportions). We computed standardised differences to compare baseline characteristics of individuals with and without metabolic syndrome and applied a threshold of 0.1 to define a meaningful difference [24].

We estimated the association between metabolic syndrome at baseline and OA incidence using flexible parametric survival models (using Stata's “stpm2” command) with 5 degrees of freedom (knots placement at 0^th^, 25th, 50th, 75th, 100th percentile), which do not assume a specific data distribution but use restricted cubic splines to model the log cumulative hazard. From the models, we estimated hazard ratios (HRs) with 95 % confidence intervals (CIs). Time in years from the baseline until the occurrence of any of the events of interest (outcome or censoring) was used as the time scale. To account for possible non-proportional hazards, we estimated models with time-dependent coefficients for metabolic syndrome with up to 3 degrees of freedom and found that a proportional hazard model was the preferred model based on the Bayesian information criterion (BIC) and Wald test of time-dependent coefficients (p-values were >0.05).

We conducted our analyses in four steps. First, we estimated a model with no adjustment for any covariates (crude model). We then added the following covariates in each step: sex and age (model 1), model 1 + education + immigration status + marital status (model 2) and finally, model 2 + physical activity + diet score (model 3). Changes in each step indicate to what extent the association between metabolic syndrome and OA incidence is assumed to be influenced by these covariates. We also conducted a sensitivity analysis excluding individuals with any pain.

### 2.8 Secondary analyses

We conducted a series of secondary analyses.

1. We conducted joint-specific analyses for knee (ICD-10 code: M17) and hip (ICD-10 code: M16) OA as our outcomes to explore the possible differences in the associations between metabolic syndrome and OA type (crude, model 1–3).
2. We explored the associations between each specific metabolic syndrome component with OA incidence adjusted for all confounders (i.e. components were studied in separate models and adjusted for confounders reported in model 3).
3. We studied the contribution of elevated WC on the risk of OA incidence by creating a variable with five categories: 1. No metabolic syndrome (i.e. <3 metabolic syndrome components) and no elevated WC, 2. elevated WC and no other metabolic syndrome component, 3. elevated WC and 1 other metabolic syndrome component, 4. metabolic syndrome (i.e. >2 metabolic syndrome components) and no elevated WC and 5. metabolic syndrome, including elevated WC.
4. We studied the association between the count of metabolic components using a four-category exposure (any one component, any two components, any three components, any four or five components [pulled together due to the low number of individuals with five components]).
5. We substituted WC with BMI≥30 as a criterion to define metabolic syndrome and repeated the main analysis as well as secondary analysis one to three with this new definition.

## 3 Results

A total of 10,633 individuals were included. Of these, 94 (0.9 %) were excluded because of unknown metabolic syndrome status. This resulted in a final sample of 10,539 individuals with a mean (standard deviation [SD]) age of 59.0 (8.7) and 49.2 % females (Table 1). In total, 5330 (50.6 %) individuals had metabolic syndrome, while 605 (5.7 %) did not have any metabolic syndrome components and 597 (5.7 %) had all five components (S2 Table). The most common combination of metabolic syndrome components was elevated WC, blood pressure and fasting glucose (19.5 % of the sample). Those with metabolic syndrome were older, more often males, had lower educational attainment, and lower diet and physical activity scores compared with those without metabolic syndrome.

**Table 1:** Characteristics at baseline.

	All	No metabolic syndrome	Metabolic syndrome	Absolute standardised difference
N	10,539	5209	5330	
Female, n (%)	5182 (49.2)	2983 (57.3)	2199 (41.3)	0.32
Age [years], mean (SD)	59.0 (8.7)	56.7 (8.5)	61.2 (8.2)	0.53
Level of education, n (%)				
≤9 years of education	946 (9.0)	325 (6.2)	621 (11.7)	0.19
10–12 years of education	3668 (34.8)	1628 (31.3)	2040 (38.3)	0.15
≥13 years of education	5925 (56.2)	3256 (62.5)	2669 (50.1)	0.25
Born in Sweden, n (%)	9280 (88.1)	4595 (88.2)	4685 (87.9)	0.01
Marital status, n (%)				
Never married	1874 (17.8)	1077 (20.7)	797 (15.0)	0.15
Previously married	2283 (21.7)	1084 (20.8)	1199 (22.5)	0.04
Married	6382 (60.6)	3048 (58.5)	3334 (62.6)	0.08
Diet score [0–8], mean (SD)	4.0 (1.7)	4.2 (1.7)	3.9 (1.7)	0.22
Body mass index [kg/cm^2^], mean (SD)	25.9 (3.7)	24.1 (2.9)	27.6 (3.5)	1.08
18–24.9, n (%)	4579 (44.0)	3455 (66.8)	1124 (21.4)	1.03
25–29.9, n (%)	4515 (43.3)	1540 (29.8)	2975 (56.7)	0.57
≥30, n (%)	1325 (12.7)	178 (3.4)	1147 (21.9)	0.58
Physical activity [1–7], mean (SD)	4.1 (1.4)	4.4 (1.4)	3.9 (1.3)	0.33
Elevated waist circumference, n (%)	6913 (65.6)	2024 (38.9)	4889 (91.7)	1.34
Elevated triglycerides, n (%)	1618 (15.4)	82 (1.6)	1536 (28.9)	0.82
Reduced HDL-C, n (%)	3004 (28.5)	384 (7.4)	2620 (49.2)	1.05
Elevated blood pressure, n (%)	7397 (70.2)	2485 (47.7)	4912 (92.2)	1.11
Elevated fasting glucose, n (%)	7360 (69.9)	2479 (47.6)	4881 (91.8)	1.10

N/n: number; SD: standard deviation; HDL-C: high-density lipoprotein cholesterol.

**Fig. 2:**
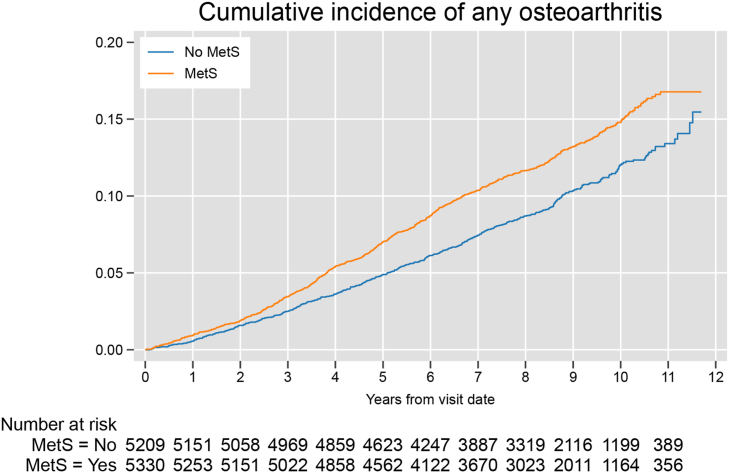
Cumulative incidence of any osteoarthritis diagnosis over the study period.

Overall, the cumulative incidence of OA and the at-risk population at each calendar year is reported in Fig. 2. During a median follow-up of 8.6 years, there were 1167 incident OA diagnoses. Among individuals with metabolic syndrome, there were 659 incident OA diagnoses over 41,860 persons per year (i.e. person-years) (300 knee OA over 43,407 persons per year and 176 hip OA over 43,961 persons per year) corresponding to an incidence rate of 15.7 (95 % CI: 14.6, 17.0) per 1000 persons per year. The corresponding figures in those without metabolic syndrome were 508 incident OA diagnoses over 42,424 persons per year of follow up (230/43,607 persons per year for knee OA, 132/43,951 persons per year for hip OA), resulting in an incidence rate of 12.0 (95 % CI: 11.0, 13.1) per 1000 persons per year.

### 3.1 The role of metabolic syndrome in incident OA

**Fig. 3:**
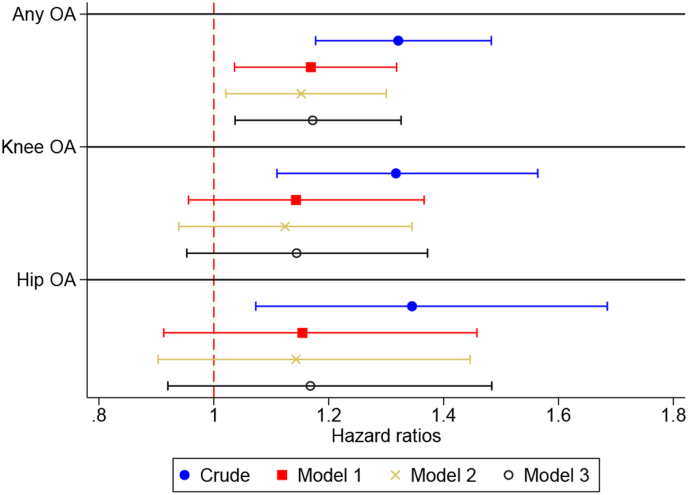
Risk of incident any OA, knee OA and hip OA in individuals with metabolic syndrome compared to individuals without. Model 1: adjusted for sex and age; Model 2: Model 1 + education + marital status + country of birth; Model 3: Model 2 + diet score + physical activity at leisure time.

The results of unadjusted survival analysis suggested that metabolic syndrome was associated with 32 % higher rates of OA incidence (HR 1.32, 95 % CI: 1.18, 1.48, Fig. 3, S3 Table). After adjustment for sex and age, the HR attenuated to 1.17 (95 % CI: 1.04, 1.32), while adjustment for further covariates did not affect this estimate. Sensitivity analysis excluding individuals with any pain led to similar results (S4 Table). Similar results were also obtained for knee and hip OA, even though adjusted models resulted in inconclusive associations (i.e., wide 95 % CI including the HR of 1), possibly due to smaller sample sizes (S3 Table).

### 3.2 The role of different metabolic syndrome components on incident OA

**Fig. 4:**
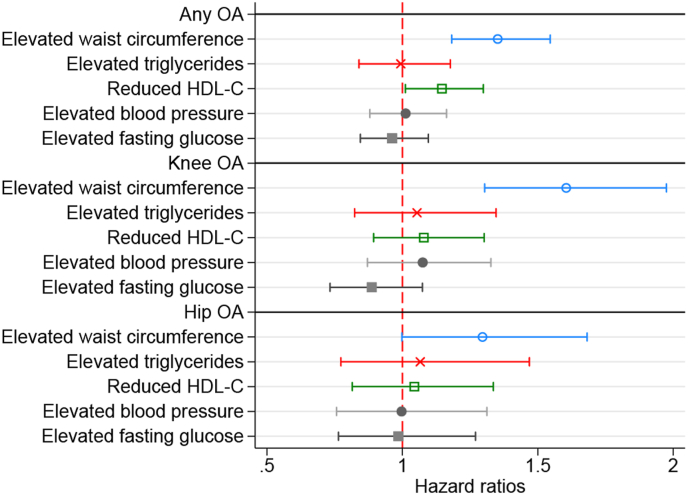
Risk of incident any OA, knee OA and hip OA in individuals with metabolic syndrome components. HDL-C: high-density lipoprotein cholesterol; Model adjusted for sex, age, education, marital status, country of birth, diet score, physical activity at leisure time (Model 3).

Exploring the associations between each metabolic syndrome component and OA incidence revealed that only elevated WC and reduced HDL-C were conclusively associated with a higher incidence of OA after adjustment for all covariates (Fig. 4, S5 Table). Moreover, the presence of elevated WC alone was associated with a similar risk of OA (1.42 [1.06, 1.90]) as metabolic syndrome with elevated WC (1.36 [1.17, 1.58]), while metabolic syndrome without elevated WC did not show a conclusive association (1.02 [0.73, 1.43]) (Table 2). Finally, the count of metabolic syndrome components (any component) showed a positive association with the risk of OA, with individuals with four to five metabolic syndrome components showing the highest risk.

**Table 2:** The role of elevated waist circumference and metabolic syndrome components count in any OA incidence.

Metabolic syndrome categorisation	Hazard Ratio (95%CIs)			
	Crude	Model 1	Model 2	Model 3
No elevated WC and <3 other metabolic syndrome components (n = 3184)	1.0 (ref)	1.0 (ref)	1.0 (ref)	1.0 (ref)
Only elevated WC (n = 481)	1.38 (1.03, 1.84)	1.40 (1.04, 1.87)	1.38 (1.03, 1.85)	1.42 (1.06, 1.90)
Elevated WC+1 another component (n = 1543)	1.42 (1.17, 1.71)	1.29 (1.07, 1.56)	1.29 (1.07, 1.56)	1.33 (1.10, 1.61)
Metabolic syndrome without elevated WC (n = 440)	1.11 (0.80, 1.54)	1.02 (0.73, 1.43)	1.01 (0.72, 1.41)	1.02 (0.73, 1.43)
Metabolic syndrome including elevated WC (n = 4889)	1.57 (1.36, 1.81)	1.33 (1.15, 1.54)	1.31 (1.14, 1.52)	1.36 (1.17, 1.58)
**Metabolic syndrome components**				
0 or any 1 component	1.0 (ref)	1.0 (ref)	1.0 (ref)	1.0 (ref)
Any 2 components	1.16 (0.97, 1.38)	1.03 (0.86, 1.23)	1.03 (0.86, 1.23)	1.04 (0.87, 1.24)
Any 3 components	1.43 (1.21, 1.69)	1.17 (0.99, 1.40)	1.16 (0.97, 1.38)	1.18 (0.99, 1.41)
Any 4 or 5 components	1.46 (1.22, 1.74)	1.21 (1.00, 1.46)	1.19 (0.98, 1.43)	1.23 (1.02, 1.49)

WC: Waist circumference; Model 1: adjusted for sex and age; Model 2: Model 1 + education + marital status + country of birth; Model 3: Model 2 + diet score + physical activity at leisure time.

### 3.3 WC vs BMI as measures of obesity

In our sample, only one individual with BMI-based obesity (out of 3595 individuals with obesity) did not have increased WC, while out of the 6,834 with increased WC, 1324 (19.4 %) had also increased BMI. Substituting WC with BMI defined obesity in the definition of metabolic syndrome, 7418 (70.4 %) individuals were classified as having no metabolic syndrome, 3108 (29.5 %) as having metabolic syndrome, while 13 (0.1 %) had missing BMI. During the follow-up, there were 393 incident cases of any OA over 24,332 persons per year (incidence rate 16.2, 95 % CI 14.6, 17.8 per 1000 persons per year) in Mets group and 773 over 59,882 persons per year (incidence rate 12.9, 95 % CI 12.0, 13.9 per 1000 persons per year) in the no metabolic syndrome group. The results of the survival analyses showed similar risks of incident any OA, knee OA and hip OA to the analyses using WC as metabolic syndrome component and when analysing BMI defined obesity as a separate component (S6-8 Table).

## 4 Discussion

Our study shows that increased WC alone leads to a similar increase in risk as when full metabolic syndrome is present, suggesting that WC is the main driver of this association and questioning whether metabolic syndrome can be considered a risk factor independently of WC. Replacing WC with BMI yielded similar results, confirming that obesity is the key factor behind this observed association.

Metabolic syndrome has been hypothesised to be a risk factor for OA acting through several mechanisms, including biomechanical joint overload due to high body mass, proinflammatory mechanisms mediated by adipokines, hyperglycaemia, and elevated levels of oxidised low-density lipoprotein, as well as by subchondral ischemia mediated by hypertension [2,25,26]. Despite the plausible causal links between metabolic syndrome and OA, epidemiological studies have reported contrasting associations between metabolic syndrome and risk of incident OA, likely due to differences in methodology, especially regarding incident OA definition and adjustments [[7], [8], [9], [10], [11], [12], [13], [14], [15]]. A 2020 systematic review and meta-analysis of prospective cohort studies examined whether metabolic syndrome is a risk factor for knee OA independent of BMI and concluded that, when analysed as a dichotomous exposure, metabolic syndrome is not an independent risk factor [27].

Disentangling the metabolic effect of metabolic syndrome from the biomechanical overload imposed by obesity is of great interest. As shown by the review from Nie and colleagues, many studies used adjustment for BMI to isolate these effects, resulting in attenuated or null associations and leading to the conclusion that metabolic syndrome is not associated with OA independent of BMI [9,11,27]. The interpretation of such results is, however, not straightforward and it is complicated by central obesity (typically measured by waist circumference, WC) being one of the metabolic syndrome components while at the same time being strongly correlated to BMI. This is particularly true when both measures are dichotomised to define obesity. In our sample, all but one individual with BMI ≥30 also had elevated WC. As a result, it becomes difficult to separate metabolic from mechanical effects by a simple adjustment. A very large sample size may, however, allow for the distinction of the effect of even highly correlated variables. This could explain why only the studies by Zhang et al. (300,000+ individuals) and Hussain et al. (20,000+ individuals, but using surgery to define incident OA) found associations between metabolic syndrome or WC and OA that persisted after adjusting for BMI [7,15]. We thus believe that null associations of metabolic syndrome with OA after adjustment for BMI should be interpreted with caution, especially in small samples and not necessarily lead to the conclusion that metabolic syndrome is not associated with incident OA. Rather, the attenuation of the association following adjustment for BMI should be interpreted as obesity (WC or BMI defined) driving the observed association between metabolic syndrome and OA. This can also be concluded from our secondary analysis showing that WC is the only metabolic syndrome component consistently associated with increased risk of OA, and that obesity alone (WC or BMI defined) leads to an increased risk of OA comparable to full metabolic syndrome, overshadowing other metabolic syndrome component combinations. This finding may have important clinical implications since it suggests that WC (or BMI) may be a better proxy to understand whether a person is at elevated risk of developing OA than metabolic syndrome.

A previous study observed that the accumulation of metabolic factors, including obesity/overweight, diabetes, and hypertension, was associated with more pain before and after an exercise-based first-line intervention [28]. Our present secondary analysis studying the association between the count of metabolic syndrome components and risk of developing OA showed an increased risk when three or four components were present. While these results may be interpreted as increased metabolic burden (i.e. more metabolic conditions present) being associated with OA, we believe that increased WC is the driver behind the observed pattern as the prevalence of individuals with WC was 27 % in those with one component, and grew to 54 %, 88 % and 96 % in the groups with respectively two, three and four components (S2 Table). At the same time, individuals with multiple conditions may also have a higher WC and BMI and have more contact with healthcare—which together can explain the higher risk of incident OA associated with the presence of multiple metabolic syndrome components. Considering the overwhelming importance of WC, future studies interested in disentangling the effect of single components or cumulative metabolic burden should avoid dichotomisation. In this context, our isolated finding that HDL-C was independently associated with increased risk of OA should be interpreted with caution and further explored.

In our study, metabolic syndrome was similarly associated with OA of the knee and the hip, while WC appeared to be more strongly associated with knee OA than hip OA, despite joint-specific estimates having lower precision from reduced sample sizes, resulting in uncertain associations. Some studies have suggested that metabolic syndrome is associated primarily with knee OA but not hip OA [10,15]. These studies used joint replacement, which combines both incidence and progression of the disease, making comparisons with studies using OA diagnosis as an outcome difficult. Nonetheless, evidence shows that obesity and other lifestyle factors appear to be more strongly related to knee than hip OA (which instead appears to have a higher heritability), both when defined as incident joint replacement and clinical diagnosis [[29], [30], [31]]. These findings could explain the stronger link between metabolic syndrome and knee OA when considering the leading role of obesity in the metabolic syndrome association with OA. To clarify the association between metabolic syndrome and incident OA in different joints, further studies with an adequate sample size are needed. This is particularly important for hand OA, where the biomechanical effects of obesity can be partially ruled out.

Our study has several strengths and limitations. One of the key strengths is the use of a large sample size, free of OA symptoms at baseline, with OA confirmed through register-based diagnoses and an up to 11-year follow-up period. Moreover, metabolic syndrome was defined through a combination of laboratory analysis, medication prescription and clinical diagnosis, thus reducing the risk of misclassification. However, there are also some limitations. Metabolic syndrome components were assessed only at baseline, meaning individuals who developed metabolic syndrome during the study period were included in the non-metabolic syndrome group, potentially with a pre-metabolic syndrome status, which could be hypothesised to impact OA risk. If pre-metabolic syndrome is similarly associated with OA as metabolic syndrome, we might have underestimated the risk of OA. In that regard, metabolic syndrome and its components might be better studied on a continuous scale or in several categories. Moreover, despite OA diagnosis in the Swedish registers having high positive predictive values, potential misclassification of the outcome (e.g. individuals at the early stages of the disease remain undiagnosed) persists, also due to individuals with joint pain and OA potentially not seeking care, and may bias the estimates towards the null [20]. Another limitation is that the cohort consisted of volunteers (usually healthier than the general population) with a mean age of 59 years, predominantly of European ancestry, which limits the possibility of generalising the results in other populations. Similarly, our sample was mainly composed of individuals of European ancestry (∼80 %), who predominantly developed knee or hip OA, thus limiting the generalisability to populations of different ancestry and to different joints. Finally, the observational nature of the study limits the possibility to infer on the mechanisms (i.e. causal link) behind the observed associations and, despite accounting for known confounders, residual confounding may still be present (e.g. from genetic susceptibility, previous injuries). Additionally, individuals with multiple health conditions tend to consult healthcare professionals more often, which may increase the likelihood of receiving further diagnoses.

## 5 Conclusions

The presence of increased WC or BMI appears to be the main driver behind the observed association between metabolic syndrome and OA. In our present study, metabolic syndrome without increased WC was not associated with an increased risk of OA, while WC alone led to an increase in the risk of incident OA equal to that of metabolic syndrome with increased WC. These findings suggest that in middle-aged and older adults without joint pain, assessing WC or BMI could provide a simple and effective way for identifying individuals at a higher risk of OA development. Consequently, monitoring and managing obesity within the context of metabolic syndrome is crucial for mitigating future OA risk.

## Author contributions

Conceptualization: Andrea Dell’Isola, Ali Kiadaliri.

Data curation: Andrea Dell’Isola, Ali Kiadaliri.

Formal analysis: Ali Kiadaliri.

Funding acquisition: Andrea Dell’Isola, Martin Englund.

Methodology: Andrea Dell’Isola, Ali Kiadaliri.

Writing – original draft: Andrea Dell’Isola.

Writing – review & editing: Andrea Dell’Isola, Karin Magnusson, Johanna Vinblad, Ali Kiadaliri, L Stefan Lohmander, Martin Englund.

## Data availability statement

The dataset includes data from EpiHealth, Swedish Drug Register, National Patient Register, provided to the researchers through a restricted-access agreement that prevents sharing the dataset with a third party or publicly. Individual-level data of patients included in this paper after deidentification are considered sensitive and will not be shared. However, the individual-level data are accessible to authorised researchers after ethical approval and application to https://www.socialstyrelsen.se/, https://registercentrum.se/and https://www.epihealth.lu.se/en/cohort.

## Declaration of funding and role of funding source

AD is supported by the Swedish Research Council (2022-01507), the Greta and Johan Kock foundation.

The funders had no role in study design, data collection and analysis, decision to publish, or preparation of the manuscript.

## Conflict of interest

ME declares consultancy for Grünenthal Sweden AB and Key2Compliance. AK and LSL act as part-time scientific advisors for Arthro Therapeutics. Other authors declare no conflict of interest.
